# Task‐based image quality and dosimetric validation of HyperSight CBCT for adaptive radiotherapy

**DOI:** 10.1002/acm2.70782

**Published:** 2026-08-28

**Authors:** Byungmoon Park, Taeyoon Kim, Kwan‐Woo Lee

**Affiliations:** ^1^ Program in Biomedical Science, Graduate School Korea University Sejong Republic of Korea; ^2^ Department of Radiation Oncology Konkuk University Medical Center Seoul Republic of Korea; ^3^ Proton Therapy Center National Cancer Center Goyang Gyeonggi‐do Republic of Korea; ^4^ Division of Semiconductor Physics Korea University Sejong Republic of Korea; ^5^ Department of Applied Physics, Graduate School Korea University Sejong Republic of Korea

**Keywords:** adaptive radiotherapy, BeO OSLD, detectability index, HyperSight, ImQuest, task‐based image quality

## Abstract

**Background:**

Cone‐beam computed tomography (CBCT) is increasingly used in adaptive radiotherapy (ART), yet the dosimetric accuracy and image quality of advanced CBCT imaging platforms—such as Halcyon HyperSight 4.0 (HH), which combines an upgraded detector panel with advanced iterative reconstruction, scatter‐correction, and metal‐artifact‐reduction algorithms—remain insufficiently characterized clinically.

**Purpose:**

To evaluate HH CBCT reconstruction algorithms for ART by assessing image quality, dose accuracy, and in vivo verification using optically stimulated luminescence dosimeters (OSLDs).

**Methods:**

Task‐based image quality was evaluated with ImQuest per AAPM TG‐233 using a Gammex 464 ACR phantom on three systems: Canon Aquilion Large Bore CT (LB, reference), TrueBeam (TB), and HH. Seven algorithms were compared (LB, TB‐FDK, TB‐iCBCT, HH‐FDK, HH‐iCBCT, HH‐Acuros, HH‐MAR), with d′ (the detectability index, a task‐based image‐quality metric per AAPM TG‐233) derived for four insert materials (polyethylene, acrylic, bone, air). A volumetric modulated arc therapy (VMAT) plan was independently re‐optimized (using identical optimization objectives/constraints as the LB reference plan) on HH‐iCBCT, HH‐Acuros, and HH‐MAR images and verified by portal dosimetry (1%/1 mm gamma), delivered to an Alderson RANDO phantom with dose measured at 15 points (2 clinical target volume [CTV], 7 organ‐at‐risk [OAR], and 6 lung points) using BeO OSLDs and compared to LB (± 5% criterion). Mean *d*′ per algorithm was compared to LB via one‐sample *t*‐tests (*p* < 0.05).

**Results:**

HH‐iCBCT (*d*′ = 4.70, *p* = 0.016), HH‐Acuros (*d*′ = 4.61, *p* = 0.005), and HH‐MAR (*d*′ = 4.71, *p* = 0.024, numerically highest) showed significant detectability improvement over LB (*d*′ = 4.42), with HH‐iCBCT most consistent across materials. Mean absolute OSLD dose errors were 1.71%, 2.09%, and 2.90%; all 15 points met the ± 5% criterion except Lt_lung2 in HH‐MAR (−5.32%).

**Conclusion:**

HH‐iCBCT demonstrated clinically acceptable image quality and dose accuracy for CBCT‐based ART, supported by in vivo BeO OSLD verification in an anthropomorphic phantom.

## 1 INTRODUCTION

In image‐guided radiotherapy (IGRT), cone‐beam computed tomography (CBCT) has become an essential tool for verifying patient positioning and assessing inter‐fraction anatomical changes.^1^, ^2^, ^3^ More recently, as the clinical importance of adaptive radiotherapy (ART) has grown, considerable research has been directed toward utilizing CBCT images not only for positional alignment but also for online treatment plan adaptation and dose calculation.^4^, ^5^, ^6^ In particular, online ART—which involves real‐time plan re‐optimization based on CBCT images acquired immediately before each treatment fraction—has emerged as a next‐generation approach capable of responding to inter‐fraction anatomical changes in real time. However, conventional CBCT reconstructed using the Feldkamp–Davis–Kress (FDK) algorithm has fundamental limitations for quantitative imaging, as its Hounsfield unit (HU) accuracy is degraded by scatter radiation, beam hardening, and high noise levels.^7^, ^8^, ^9^ To address these limitations, modern CBCT systems such as Halcyon HyperSight 4.0 (HH; Varian Medical Systems, Palo Alto, CA) integrate iterative reconstruction, scatter correction (Acuros), and metal‐artifact‐reduction (MAR) algorithms to support quantitative dose calculation.^10^, ^11^

Lustermans et al.^10^ evaluated the HU accuracy of the HH system and reported considerable improvements in image fidelity over conventional FDK‐based CBCT. However, their evaluation was limited to conventional noise‐ and contrast‐based metrics without adopting the task‐based image quality framework recommended by AAPM TG‐233. Kim et al.^11^ also confirmed improved HU accuracy with the HH system in a clinical setting. These results have stimulated interest in the direct use of HH CBCT for dose calculation in ART workflows.^12^, ^13^ Nevertheless, a rigorous evaluation of image quality using task‐based metrics that better reflect clinical detectability remains largely absent from the literature.

Since iterative reconstruction algorithms exhibit nonlinear and object‐dependent noise characteristics, conventional contrast‐to‐noise ratio (CNR) or standard deviation (SD)‐based metrics do not adequately reflect true clinical performance.^14^ According to the AAPM Task Group 233 (TG‐233) report, a task‐based evaluation framework using the noise power spectrum (NPS), task transfer function (TTF), and detectability index (*d*′) is recommended and has been widely validated for CT and CBCT performance assessment.^14^, ^15^, ^16^, ^17^, ^18^ ImQuest (Duke University, Durham, NC), the reference software tool recommended by TG‐233, implements the NPS–TTF–d′ framework with validated algorithms and was therefore chosen as the analysis platform in our study. Lassot‐Buys et al.^19^ applied this framework to compare FDK and iterative CBCT algorithms on the Halcyon V2.0, reporting that iterative reconstruction significantly reduced noise and improved d′ across all acquisition modes. However, their study did not evaluate the Acuros scatter‐correction and MAR algorithms integrated in HH 4.0, nor did it assess dose calculation accuracy, leaving a critical gap for ART applications that require both image quality and dosimetric reliability.

Regarding dose calculation accuracy, the phantom‐based study using HH CBCT by Nelson et al.^20^ showed that, when a CBCT‐specific HU‐to‐density calibration curve was applied, the mean dose difference relative to the CT simulator remained within 1%. Furthermore, the 3D gamma pass rate using a 1%/1 mm criterion exceeded 90% for both pelvic and head‐and‐neck intensity‐modulated radiation therapy (IMRT) treatment plans. However, that study relied on single‐point absolute dose verification using an ion chamber and did not include multi‐point in vivo dose measurements in an anthropomorphic phantom, leaving the spatial distribution of dose errors across heterogeneous anatomical regions uncharacterized. This limitation was mitigated by using optically stimulated luminescence dosimeters (OSLDs), as described below.

In vivo dosimetry, which directly compares treatment planning system‐calculated doses with those actually delivered, is a cornerstone of clinical quality assurance guidelines issued by AAPM and International Atomic Energy Agency (IAEA).^21^ OSLDs are increasingly favored for in vivo verification in complex radiotherapy owing to their high accuracy, reusability, and stable response.^21^ Among available OSLD materials, beryllium oxide (BeO; effective atomic number Z_eff_ ≈ 7.2) offers near‐tissue equivalence and high sensitivity, making it particularly well suited for multipoint dose measurements in heterogeneous anatomical environments such as anthropomorphic phantoms. Additionally, Alvarez et al.^22^ reported a measurement uncertainty of 1.6% (1σ) in a remote dosimetry audit program, validating sufficient precision to support CBCT‐based ART dose verification. Therefore, BeO OSLDs were used for in vivo dosimetric verification in this study.

In this study, we evaluated the feasibility of HH reconstruction algorithms for clinical ART through an integrated assessment comprising: (1) TG‐233‐compliant task‐based image quality evaluation across all four insert materials (polyethylene, acrylic, bone, and air) using ImQuest, (2) electronic portal imaging device (EPID)‐based portal dosimetry, and (3) in vivo absolute dose verification at 15 anatomical points of interest in the Alderson RANDO anthropomorphic phantom using BeO OSLDs.

## 2 MATERIALS AND METHODS

### 2.1 Imaging systems and acquisition parameters

**FIGURE 1:**
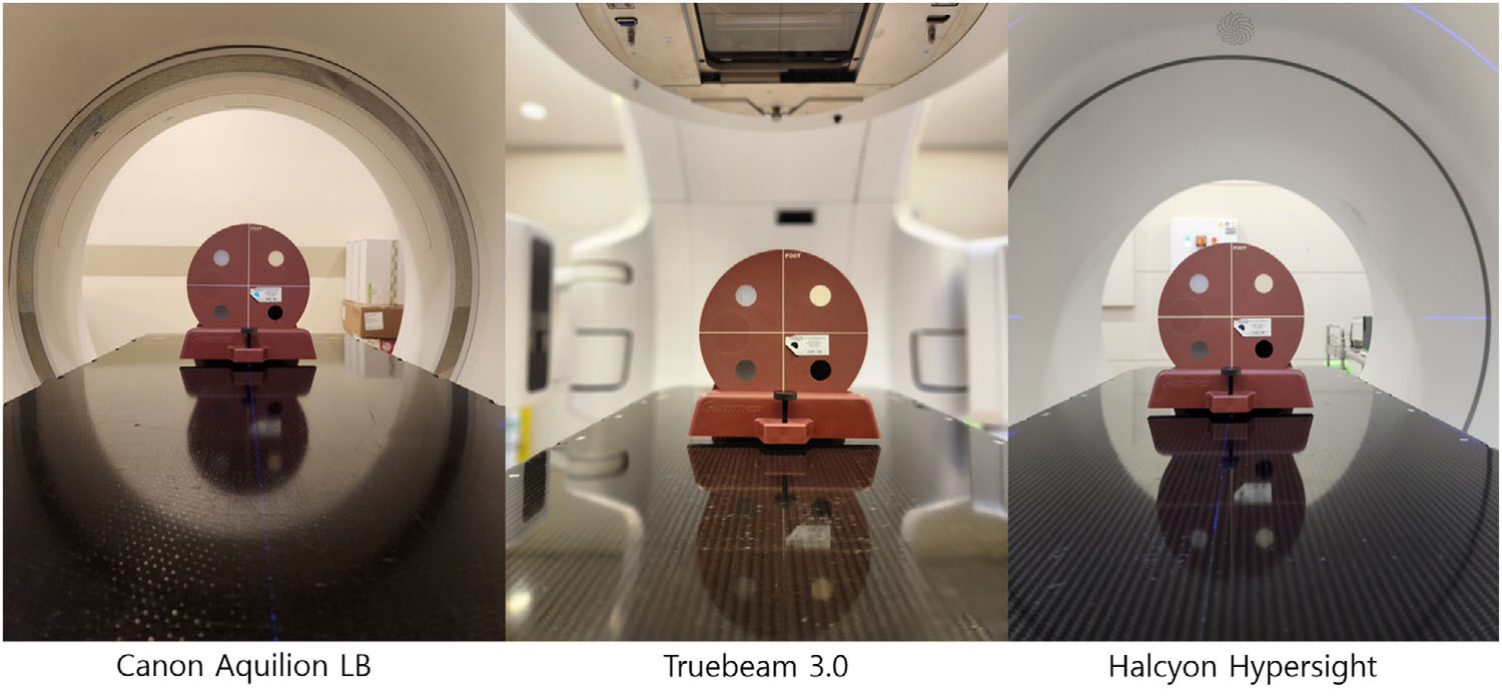
Setup photographs for image acquisition using the Gammex 464 ACR phantom on each imaging system.

The Gammex 464 ACR CT phantom (Sun Nuclear Corporation, Melbourne, FL) was used in this study. Three imaging systems were compared: (1) Canon Aquilion Large Bore (LB) planning CT, (2) TB CBCT, and (3) HH CBCT. Seven reconstruction algorithms were evaluated: LB (baseline reference), TB‐FDK, TB‐iCBCT, HH‐FDK, HH‐iCBCT, HH‐Acuros, and HH‐MAR. LB was acquired at 120 kVp and 250 mAs. TB and HH CBCT were acquired at 125 kVp, with tube charge of 247.5 mAs and 245.4 mAs, respectively; rather than using each system's clinical default protocol, these exposure settings were manually adjusted to approximate the LB acquisition condition (120 kVp, 250 mAs), so that inter‐system image‐quality differences would primarily reflect reconstruction‐algorithm performance rather than differences in acquisition exposure. All images were reconstructed with a slice thickness of 2 mm, a field of view (FOV) of 40 cm, and a 512 × 512 matrix. For each CBCT modality (excluding LB), three repeated acquisitions were performed using each reconstruction algorithm (Figure 1). All HH acquisitions used the CBCT‐for‐planning (dose‐calculation‐capable) acquisition mode, and the mAs level was confirmed to meet the vendor's minimum‐exposure requirement for HH‐on‐Halcyon dose‐calculation use. No convolution filter or noise‐suppression parameters were varied beyond the matched exposure setting described above.

### 2.2 Task‐based image quality analysis using ImQuest

**FIGURE 2:**
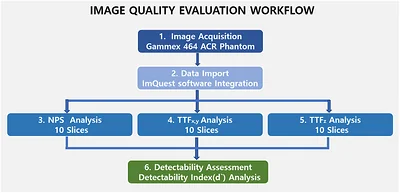
Workflow of the quantitative image quality evaluation using the Gammex 464 ACR phantom. The process includes data acquisition, software integration with ImQuest, and detailed analysis of noise power spectrum (NPS), in‐plane task transfer function (TTF), longitudinal TTF, and the overall detectability index d′.

Image quality evaluation was performed using ImQuest (Duke University, Durham, NC) in accordance with TG‐233 recommendations. ImQuest is the task‐based image quality analysis tool recommended TG‐233 and has been validated in several studies.^14^, ^15^, ^19^ The alpha build used in this study was provided by Duke University for research purposes. Analysis was performed across all four insert materials—polyethylene, acrylic, bone, and air—to assess material‐dependent variations in reconstruction algorithm performance (Figure 2).

**Noise Power Spectrum (NPS)**: The NPS quantifies the frequency‐domain noise distribution of the image signal within a region of interest (ROI), defined as ^14^:

$$NPS\left(f_{x},f_{y}\right)=\frac{1}{A}<{\left|F\left\{I\left(x,y\right)-\mu \right\}\right|}^{2}>$$

where I (*x,y*) is the image signal within the ROI, *μ* is the mean signal value, *F* denotes the two‐dimensional Fourier transform, and A is the normalization area. Multiple ROIs were placed in the homogeneous background region at the center of the phantom to minimize positional bias, without overlap between ROIs.

**Task Transfer Function (TTF)**: The TTF was derived from the Fourier transform of the line spread function (LSF), which was calculated from the edge spread function (ESF) at the insert boundary ^14^, ^15^:

$$TTF\left(f\right)=\left|F\frac{d}{dx}ESF\left(x\right)\right|$$

TTF_50_ (f_50_), defined as the spatial frequency at which the TTF decreases to 50% of its maximum value, was used as a representative indicator of system spatial resolution. Eccentricity and tilt at the insert boundary were corrected during ESF extraction to ensure stable measurements.

**Detectability Index (*d*′)**: *d*′ was calculated using the non‐pre‐whitening eye filter (NPWE) observer model, which applies an eye filter E(f) that reflects the spatial‐frequency sensitivity characteristics of the human visual system^14^, ^16^, ^23^:


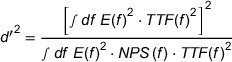

where the eye filter E(f) models the contrast sensitivity function of the human visual system by integrating both NPS and TTF. In accordance with the Rose model criterion recommended by TG‐233,^14^ a *d*′ ≥ 3.3 was defined as the clinical acceptance threshold, corresponding to a detection probability of ≥ 95% for a given task. The task used in the analysis was defined as a circular disk signal with a diameter of 20 mm.

**HU Accuracy**: Mean HU values were measured within circular ROIs (20 mm diameter) for all insert materials (polyethylene, bone, acrylic, and air). HU differences (ΔHU) were calculated relative to the LB planning CT. All ROIs were positioned at equal distances from the phantom center to ensure consistency of inter‐algorithm comparisons. A one‐sample t‐test was applied to compare the mean *d*′ values of each reconstruction algorithm (averaged across four materials, *n* = 3 repeated acquisitions) against the LB planning CT reference value (μ_0_). Comparisons were performed using the four‐material mean *d*′ values per replicate (*n* = 3), with statistical significance defined as *p* < 0.05.

### 2.3 Dosimetric evaluation: portal dosimetry and in vivo BeO OSLD verification in the alderson radiation therapy anthropomorphic phantom

A volumetric modulated arc therapy (VMAT) treatment plan was generated in Eclipse v18.0 using LB planning CT as the reference image (6 MV flattening filter free [FFF], dual‐arc, prescribed dose 250 cGy; monitor units of 875.3–879.7 MU (the total plan MU across the LB reference plan and the three independently re‐optimized HH plans described below, a spread of < 0.5%), with Acuros XB v13.5 as the dose calculation algorithm. To emulate a realistic online‐ART re‐planning workflow, the VMAT plan was independently re‐optimized (rather than simply forward‐recalculated with a fixed MLC/MU sequence) on each of the HH‐iCBCT, HH‐Acuros, and HH‐MAR CBCT datasets, using the same beam geometry, optimization objectives, and dose–volume constraints as the LB reference plan; consequently, the final MLC sequence and total MU differed slightly between the reference plan and each independently re‐optimized HH plan. The LB‐derived HU‐to‐electron density (HU‐to‐ED) calibration curve, pre‐imported into Eclipse v18.0, was applied uniformly across all CBCT reconstruction algorithms, reflecting our institution's current online‐ART workflow in which a single pre‐validated calibration curve, rather than an algorithm‐specific curve requiring separate commissioning for each reconstruction, is used for time‐sensitive online re‐planning. EPID‐based portal dosimetry was performed using a 1%/1 mm gamma criterion, recording the gamma pass rate, mean gamma value, and mean dose difference as a pre‐treatment, delivery‐oriented fluence verification of the re‐optimized MLC/MU sequences; the direct, absolute point‐dose validation of CBCT‐based dose calculation is provided separately by the in vivo BeO OSLD measurements described below.^24^, ^25^

**FIGURE 3:**
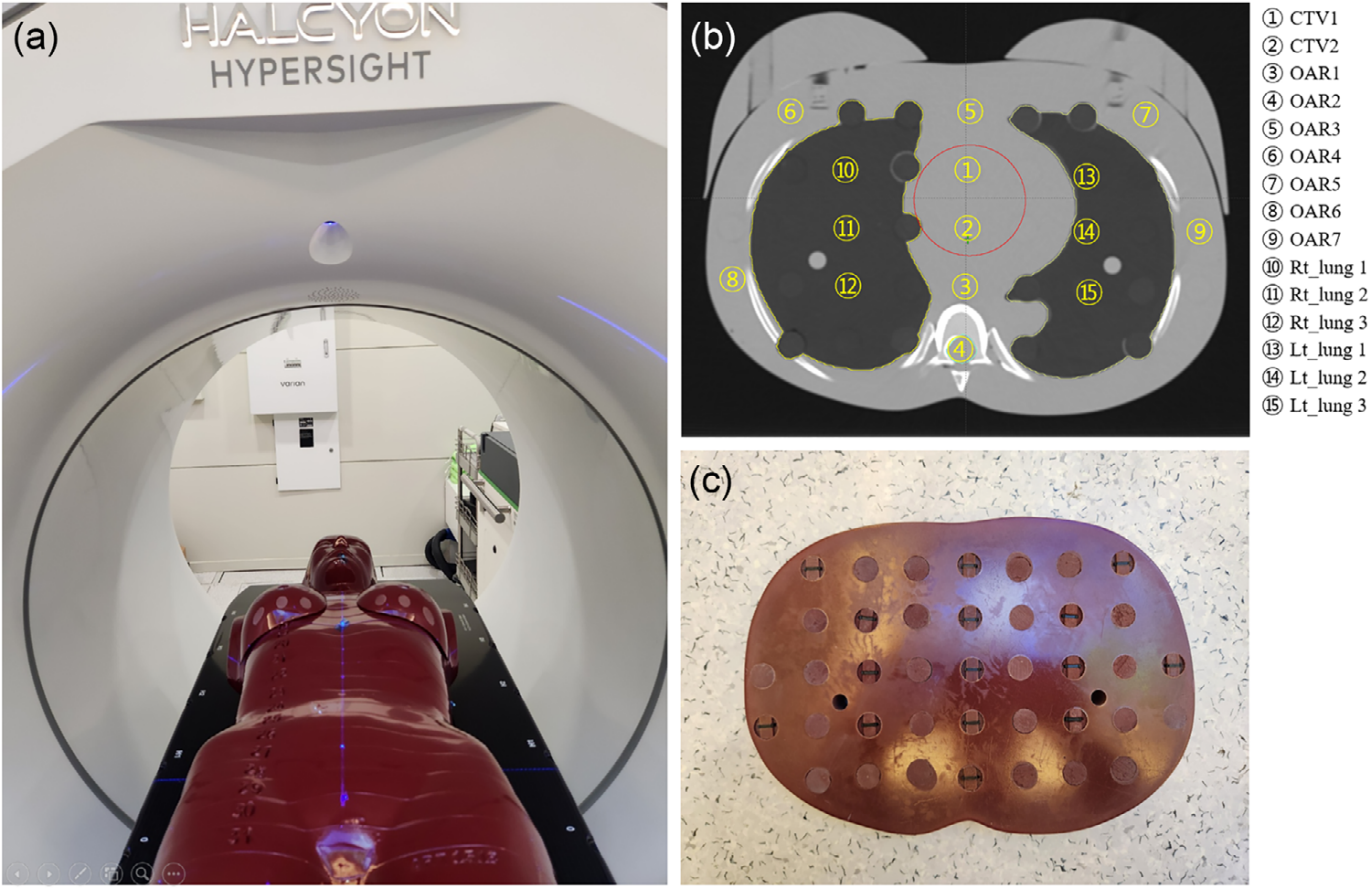
(a) Photograph of the RANDO anthropomorphic phantom positioned on the Halcyon HyperSight system for in vivo dosimetric verification. (b) Axial CT image showing the 15 anatomical points of interest (2 CTV, 7 OAR, and 6 lung points) selected for BeO OSLD placement. (c) Photograph of a transverse phantom slice showing the cavities in which the BeO OSLDs were inserted.

**In Vivo BeO OSLD Dosimetric Verification in the Alderson Radiation Therapy Anthropomorphic Phantom**: The RANDO anthropomorphic phantom (Alderson Research Laboratories, Stanford, CA) was used for in vivo dosimetric verification. BeO OSLDs (myOSLDchip, RadPro, Germany; outer dimensions 9.5 × 10 × 2 mm, active element 4.7 × 4.7 × 0.5 mm, measurement range of ∼1 µGy to 10 Gy) were inserted at 15 anatomical points of interest covering varying tissue densities and treatment positions: 2 clinical target volume (CTV) points, 7 organ‐at‐risk (OAR) points, and 6 lung points (Figure 3). The same VMAT treatment plan was delivered for each reconstruction algorithm (HH‐iCBCT, HH‐Acuros, and HH‐MAR), with three repeated irradiations per algorithm. After irradiation, BeO OSLDs were read using a RadPro OSLD reader, and the mean of three measurements was taken as the representative dose. OSLDs were calibrated under 6 MV FFF beam conditions. Since the effective atomic number of BeO (Z_eff_ ≈ 7.2) is close to that of soft tissue, energy dependence is low and angular dependence is expected to be negligible in the multi‐directional VMAT irradiation geometry. Relative dose differences (%) between each HH algorithm and LB‐measured doses were evaluated against the clinical acceptance criterion of ± 5%.

### 2.4 Use of artificial intelligence tools

An AI‐based language model (Claude, Anthropic) was used to assist with language editing of the manuscript and to generate figures based on the measured data. All scientific content, data analysis, and interpretations were performed and verified by the authors.

## 3 RESULTS

### 3.1 Task‐based image quality assessment

**FIGURE 4:**
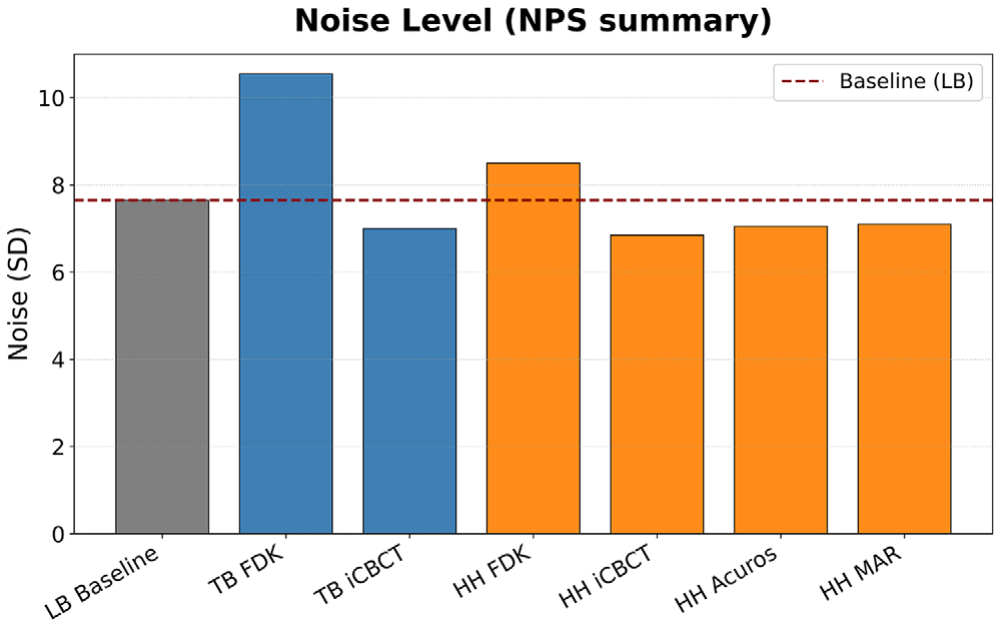
Comparison of image noise levels (NPS summary) across different imaging systems and reconstruction algorithms. Image noise, expressed as the SD, was evaluated using ImQuest software. The red dashed line denotes the reference noise level measured from the Canon Aquilion LB planning CT.

Task‐based image quality metrics derived from ImQuest analysis are summarized in Table 1. In NPS analysis, HH‐iCBCT achieved the lowest noise (SD = 6.89 HU), representing a 10.3% reduction compared with LB (7.68 HU). HH‐Acuros (SD = 7.08 HU, −7.8%) and HH‐MAR (SD = 7.10 HU, −7.6%) showed comparable noise reductions. In contrast, TB‐FDK showed the highest noise (SD = 10.56 HU), corresponding to a 37.5% increase relative to LB. HH‐FDK (SD = 8.50 HU, +10.7%) also exceeded the LB noise level (Figure 4). As shown in Table 1, HU accuracy relative to LB (ΔHU) showed systematic deviations across all CBCT algorithms, with relatively larger differences observed for bone (+47.5 to +69.2 HU) and polyethylene (+8.1 to +23.7 HU). HH‐FDK exhibited the largest HU deviations across all materials (polyethylene: +23.7 HU; bone: +69.2 HU; acrylic: +10.2 HU; air: +10.9 HU), while TB‐iCBCT and HH‐Acuros showed comparatively smaller deviations for polyethylene (+8.1 HU and +11.0 HU, respectively) and acrylic (+2.1 HU and +2.3 HU, respectively).

**TABLE 1:** Quantitative performance comparison of reconstruction algorithms relative to the LB planning CT baseline.

			*d*′ per Insert						ΔHU vs LB					
Sys.	Algo.	Overall *d*′ (mean ± SD)	PE	Acry.	Bone	Air	Noise SD (HU)	ΔNoise (%)	PE	Acry.	Bone	Air	Cohen ’s d	*p*‐value
LB	Base.	4.42 (ref)	4.34	4.42	4.50	4.41	7.68	—	—	—	—	—	—	Ref.
TB	FDK	3.05 ± 0.03	3.63	2.85	2.92	2.80	10.56	+37.5	+10.8	+2.0	+55.9	+2.6	−45.67	< 0.001^***^
	iCBCT	4.62 ± 0.07	4.61	4.57	4.78	4.53	7.00	−8.9	+8.1	+2.1	+49.7	+1.3	2.86	0.035^*^
HH	FDK	3.47 ± 0.02	3.53	3.38	3.53	3.44	8.50	+10.7	+23.7	+10.2	+69.2	+10.9	−47.50	< 0.001^***^
	iCBCT	4.70 ± 0.06	4.81	4.57	4.66	4.77	6.89	−10.3	+16.3	+11.3	+62.8	+4.9	4.67	0.016^*^
	Acuros	4.61 ± 0.02	4.66	4.64	4.49	4.64	7.08	−7.8	+11.0	+2.3	+50.2	+6.6	9.50	0.005^**^
	MAR	4.71 ± 0.08	5.41	4.31	4.46	4.65	7.10	−7.6	+11.1	+2.2	+47.5	+4.0	3.63	0.024^*^

*Note*: Overall *d*′: one‐sample *t*‐test versus LB reference mean (*μ*_0_ = 4.42), *n* = 3 replicates per algorithm. Cohen's d interpreted per Sawilowsky (2009): 0.2 = small, 0.5 = medium, 0.8 = large, 1.2 = very large, 2.0 = huge. All HH algorithms exceed the *d* = 2.0 threshold. ΔNoise (%): noise SD change relative to LB (7.68 HU). ΔHU: mean HU difference relative to LB per insert.

Abbreviations: PE, polyethylene; LB, Canon Aquilion LB planning CT (reference); TB, TrueBeam 3.0; HH, Halcyon HyperSight 4.0.

^*^ *p* < 0.05, ^**^ *p* < 0.01, ^***^ *p* < 0.001; ns = not significant. Note: *n* = 3 (df = 2); p‐values reflect limited statistical power. All six comparisons remain statistically significant after Holm‐Bonferroni correction for multiple comparisons (*α* = 0.05; see Discussion for adjusted thresholds). Cohen's d is provided as a supplementary effect size measure, independent of the *p*‐value/Holm correction above, characterizing the magnitude of each difference regardless of statistical significance.

**FIGURE 5:**
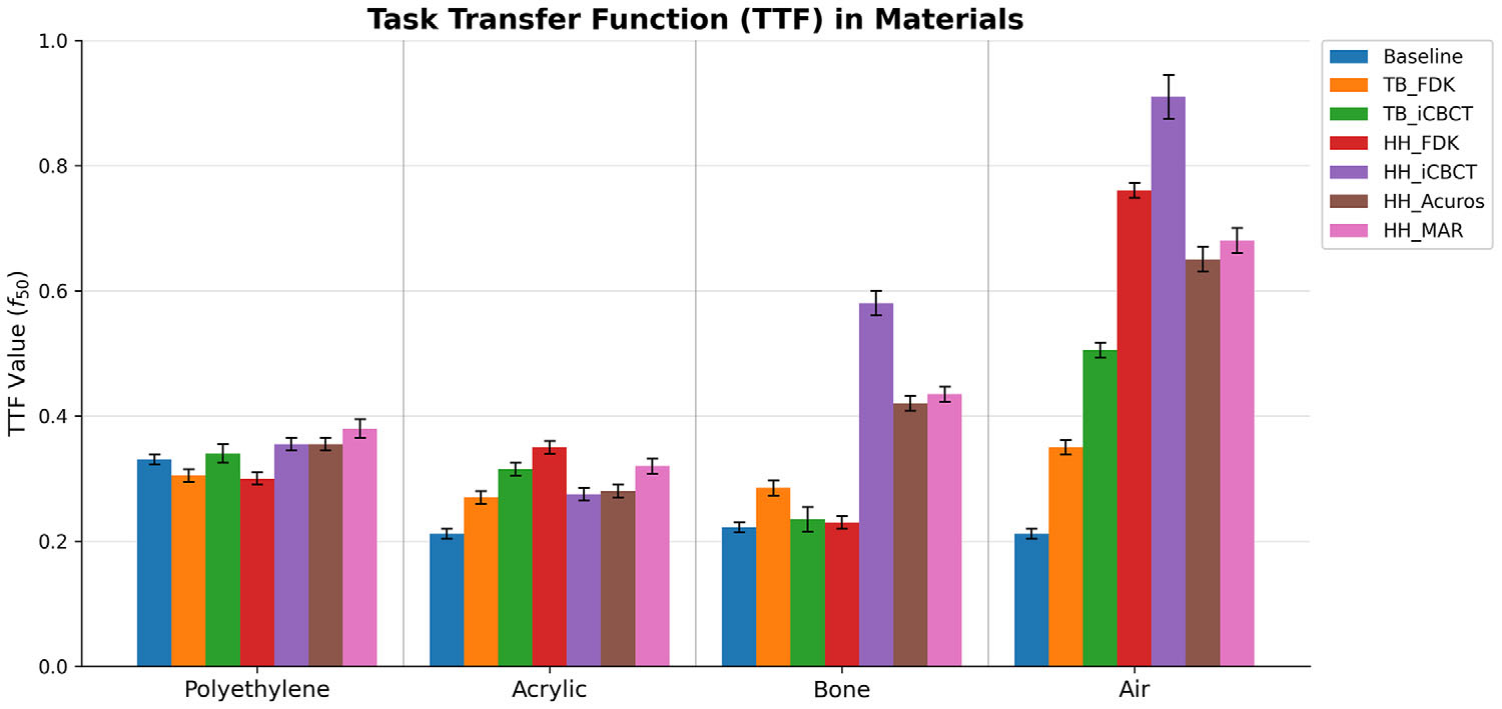
Comparison of the task transfer function at 50% (f_50_) across all four insert materials (polyethylene, acrylic, bone, and air) for each reconstruction algorithm.

In TTF analysis, f_50_ differences among algorithms were modest. HH‐Acuros and HH‐MAR tended to maintain or slightly improve f_50_ relative to LB, whereas HH‐iCBCT showed a slight reduction in f_50_, consistent with the noise–resolution trade‐off inherent to iterative reconstruction. Overall, HH‐iCBCT and HH‐Acuros achieved planning CT‐level detectability by noise suppression without substantial loss of spatial resolution, whereas HH‐FDK and TB‐FDK fell below the LB reference in task‐based image quality (Figure 5).

As given in Table 1, one‐sample t‐tests comparing reconstruction algorithms against the Canon Aquilion LB planning CT (reference value *μ*_0_ = 4.42) revealed statistically significant differences in the detectability index (*d*′). Among the HH algorithms, HH‐iCBCT showed a statistically significant 6.3% improvement over LB (*d*′ = 4.70 ± 0.06; *p* = 0.016), and HH‐Acuros also significantly exceeded the LB reference (*d*′ = 4.61 ± 0.02; *p* = 0.005). HH‐MAR recorded the numerically highest overall *d*′ (4.71 ± 0.08), which also reached statistical significance relative to LB (*p* = 0.024). Among the HH algorithms, HH‐FDK exhibited the lowest *d*′ (3.47 ± 0.02; *p* < 0.001), marginally exceeding the clinical acceptance threshold defined by the Rose criterion (*d*′ ≥ 3.3). For the TB algorithms, TB‐iCBCT significantly exceeded the LB reference (4.62 ± 0.07; *p* = 0.035), whereas TB‐FDK was significantly lower than the reference value (3.05 ± 0.03; *p* < 0.001).

**FIGURE 6:**
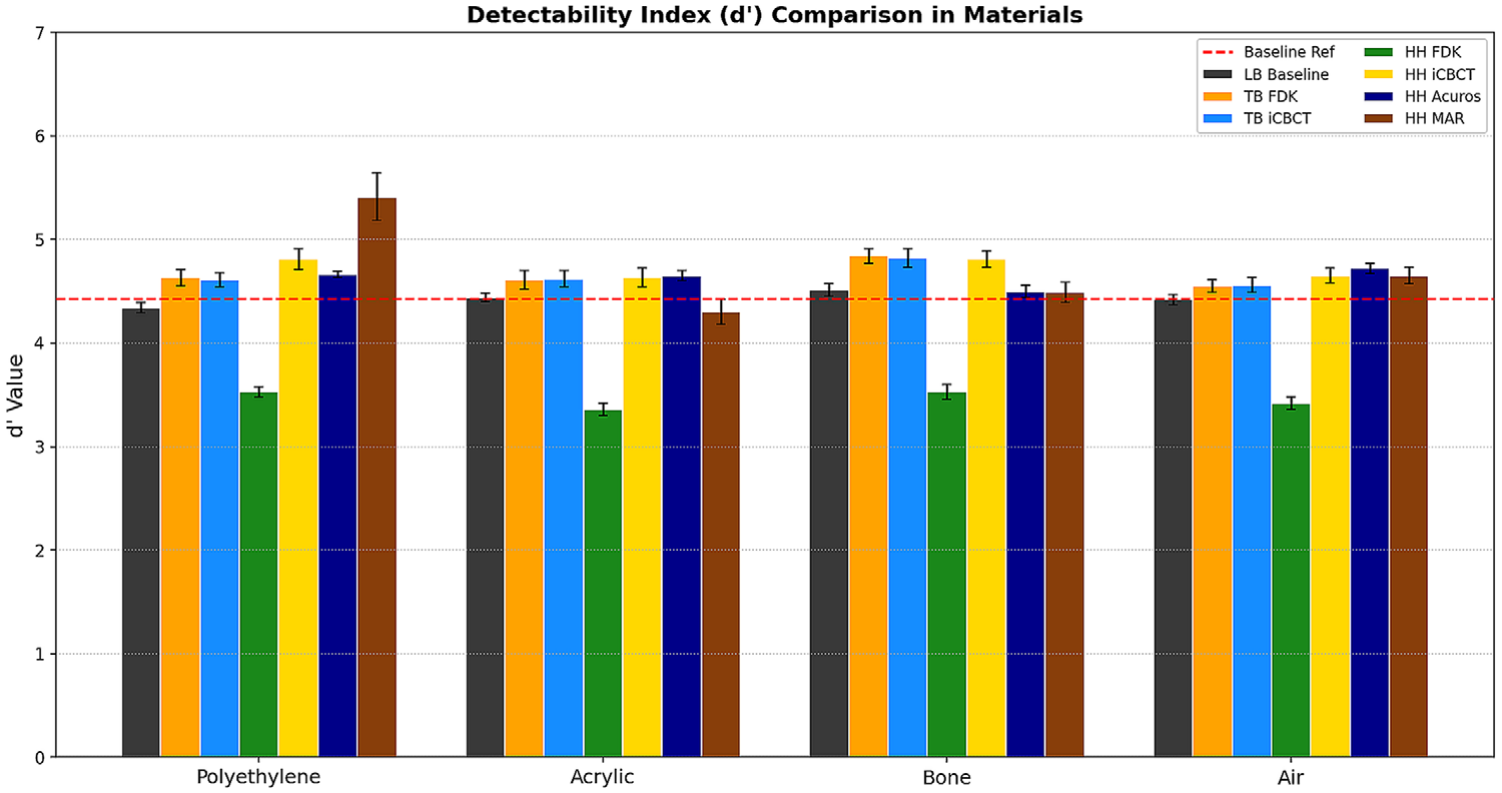
Comparison of the detectability index d′ for all four insert materials (polyethylene, acrylic, bone, and air) across imaging systems and reconstruction algorithms. The red dashed line indicates the Canon Aquilion LB baseline reference.

Material‐specific *d*′ analysis revealed material‐dependent variability in performance among the algorithms. HH‐iCBCT maintained *d*′ values that were equal to or higher than the LB reference across all four insert materials—polyethylene (*d*′ = 4.81), acrylic (*d*′ = 4.57), bone (*d*′ = 4.66), and air (*d*′ = 4.77)— indicating the most consistent detectability performance among the evaluated algorithms. HH‐MAR exhibited a notably elevated *d*′ for polyethylene (5.41, +24.6% relative to LB), while showing relatively lower values for acrylic (*d*′ = 4.31) and bone (*d*′ = 4.46), indicating material‐dependent variability. HH‐Acuros maintained *d*′ values at or above the LB reference for acrylic (*d*′ = 4.64), air (*d*′ = 4.64), and polyethylene (*d*′ = 4.66), but was marginally below the reference for bone (*d*′ = 4.49) (Figure 6).

### 3.2 Dosimetric evaluation of the Gammex 464 ACR phantom via eclipse‐calculated doses and portal dosimetry

Dosimetric verification of CBCT‐based dose calculation using the Gammex 464 ACR phantom, assessed through Eclipse v18.0 calculations and portal dosimetry, revealed that the normalized Eclipse‐calculated D95% values ranged from 97.2% to 97.5%, comparable to LB (96.9%), and the conformity index (CI) was 1.00–1.01 across all algorithms. Portal dosimetry gamma analysis (1%/1 mm criterion) yielded pass rates of 96.1% for LB, 95.0% for HH‐iCBCT, 92.9% for HH‐Acuros, and 93.3% for HH‐MAR, all meeting the clinical acceptance criterion of ≥ 90%. These results are consistent with the findings of Nelson et al.,^20^ who reported 3D gamma pass rates of ≥ 90% at the 1%/1 mm criterion (Table 2).

**TABLE 2:** Comparison of Eclipse v18.0 treatment plan dose and portal dosimetry accuracy across reconstruction algorithms using the Gammex 464 ACR phantom.

Algorithm	Mean Dose (Norm.)	CI	D95% (%)	Gamma 1%/1 mm (%)	Avg. Gamma	Total Plan MU
LB (Ref.)	100.0%	1.00	96.9	96.1	0.325	879.7
HH‐iCBCT	100.0%	1.00	97.2	95.0	0.340	875.3
HH‐Acuros	99.8%	1.00	97.5	92.9	0.360	876.7
HH‐MAR	100.0%	1.01	97.4	93.3	0.385	878.1

*Note*: CI = conformity index; D95% = dose covering 95% of CTV; Gamma 1%/1 mm (%) = gamma pass rate based on portal dosimetry (1%/1 mm criterion); Avg. Gamma = mean gamma index value from portal dosimetry analysis; Mean Dose (Norm.) = mean calculated dose normalized to the prescribed dose, expressed relative to the LB reference. Total Plan MU = total monitor units of the LB reference plan and of each independently re‐optimized HH plan (see Section 2.3); individual values fall within the 875.3–879.7 MU range reported in the text.

### 3.3 In vivo BeO OSLD dosimetric verification in the Alderson Radiation Therapy anthropomorphic phantom

**FIGURE 7:**
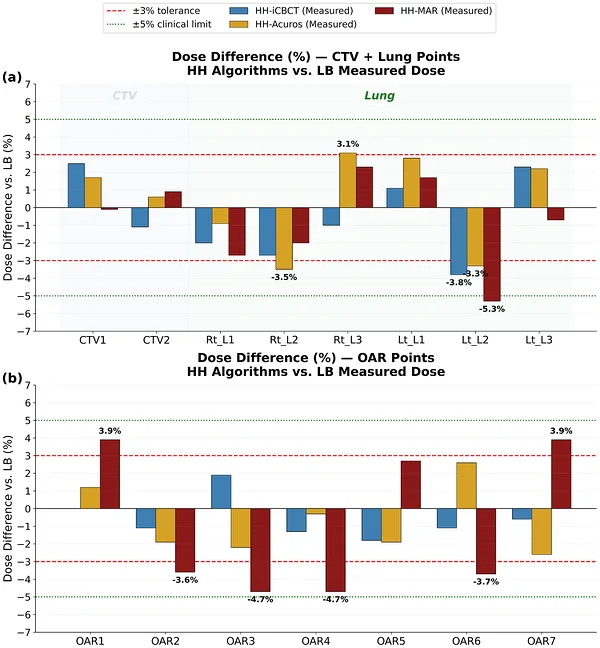
Point‐dose differences (%) relative to LB measured dose at 15 anatomical points of interest in the RANDO anthropomorphic phantom, measured with BeO optically stimulated luminescence dosimeters (OSLDs) for HH‐iCBCT, HH‐Acuros, and HH‐MAR (mean of three repetitions). Red dashed lines indicate the ± 3% tolerance, and green dotted lines indicate the ± 5% clinical limit. (a) CTV and lung points, with shaded blue and green backgrounds distinguishing the CTV points (CTV1–CTV2) and lung points (Rt_L1–Lt_L3), respectively. (b) Organ‐at‐risk (OAR) points (OAR1–OAR7). Values exceeding the ± 3% tolerance are labeled with the corresponding percentage.

Dose measurements at 15 points of interest obtained using BeO OSLDs in the Alderson Radiation Therapy anthropomorphic phantom are presented in Table 3A, 3B and Figure 7. For HH‐iCBCT, the mean absolute dose error across the 15 measurement points was 1.71% (maximum: 3.78% at Lt_lung2). HH‐Acuros yielded a mean absolute error of 2.09% (maximum: 3.52% at Rt_lung2), while HH‐MAR recorded a higher mean value of 2.90% (maximum: 5.32% at Lt_lung2). HH‐iCBCT and HH‐Acuros satisfied the clinical acceptance criterion of ± 5% at all 15 points. Notably, the Lt_lung2 point for HH‐MAR (−5.32%) exceeded the clinical tolerance limit of ± 5%. On the other hand, CTV1 and CTV2 showed good agreement within 2.5% across all reconstruction algorithms. Greater variability was observed at lung measurement points (Rt_lung and Lt_lung), likely reflecting the sensitivity of CBCT HU reconstruction to the low electron density and heterogeneous tissue composition of lung parenchyma. Dose errors at the OAR points (OAR1–OAR7) averaged approximately 3% across all algorithms, remaining within clinically acceptable ranges. As a whole, HH‐iCBCT and HH‐Acuros showed generally concordant dose trends, whereas HH‐MAR exhibited relatively larger deviations at lung and selected OAR measurement points.

**TABLE 3A:** Comparison of BeO OSLD measured doses at 15 anatomical points of interest in the Alderson Radiation Therapy anthropomorphic phantom across HH reconstruction algorithms **(unit: cGy)**.

Meas Point	LB Plan Dose (cGy)	LB Measured (cGy)	HH iCBCT Measured (cGy)	HH Acuros Measured (cGy)	HH MAR Measured (cGy)
CTV1	239.3	245.03 ± 2.81	251.27 ± 1.82	249.47 ± 0.85	244.53 ± 2.29
CTV2	236.7	243.30 ± 1.23	240.43 ± 0.71	244.80 ± 1.10	245.53 ± 0.85
OAR1	111.1	111.73 ± 1.80	111.70 ± 0.72	113.10 ± 0.26	116.13 ± 0.21
OAR2	74.5	75.80 ± 0.72	74.97 ± 0.42	74.37 ± 1.22	73.10 ± 0.46
OAR3	142.0	142.43 ± 1.44	145.30 ± 0.72	139.37 ± 0.35	135.70 ± 0.70
OAR4	74.5	76.03 ± 1.50	75.03 ± 0.40	75.80 ± 1.14	72.43 ± 0.78
OAR5	47.3	48.10 ± 0.79	47.13 ± 0.49	47.00 ± 0.17	49.40 ± 1.35
OAR6	42.3	42.87 ± 0.40	42.33 ± 1.20	44.00 ± 0.75	41.30 ± 0.30
OAR7	43.5	44.90 ± 0.72	44.57 ± 0.31	43.73 ± 0.35	46.63 ± 0.42
Rt_lung1	98.2	98.80 ± 0.52	96.87 ± 0.57	98.23 ± 0.42	95.93 ± 0.51
Rt_lung2	100.3	103.03 ± 0.84	100.03 ± 0.80	99.40 ± 0.62	100.97 ± 0.61
Rt_lung3	61.7	61.87 ± 0.64	61.17 ± 0.83	63.77 ± 0.61	63.37 ± 0.70
Lt_lung1	79.5	80.53 ± 1.10	81.47 ± 0.65	82.87 ± 1.63	82.00 ± 0.72
Lt_lung2	103.1	105.83 ± 1.20	101.83 ± 0.65	102.33 ± 0.55	100.20 ± 0.10
Lt_lung3	77.7	79.30 ± 1.04	81.33 ± 0.68	81.20 ± 0.87	78.73 ± 1.35
Max Diff. (%)	—	3.22 (OAR7)	−3.78 (Lt_lung2)	−3.52 (Rt_lung2)	−5.32 (Lt_lung2)
Min Diff. (%)	—	0.28 (Rt_lung3)	−0.03 (OAR1)	−0.30 (OAR4)	−0.20 (CTV1)

*Note*: LB = Canon Aquilion LB planning CT (reference); HH = Halcyon HyperSight; CTV = clinical target volume; OAR = organ at risk. Dose data are expressed as mean ± standard deviation (SD) from three independent replicates. Max Diff. and Min Diff. represent the maximum and minimum relative dose differences (by absolute magnitude). For LB, the difference is relative to the Plan Dose; for HH algorithms, the difference is relative to the LB Measured Dose.

Because the dose distribution of each independently re‐optimized HH plan was retained in the treatment planning system, the Eclipse‐calculated dose at each of the 15 OSLD point locations was additionally extracted for HH‐iCBCT, HH‐Acuros, and HH‐MAR and compared directly with the corresponding OSLD‐measured dose, without requiring any additional irradiation. This calculated‐versus‐measured comparison, presented alongside the original LB‐measured‐referenced comparison above (Table 3A, 3B), isolates the accuracy of CBCT‐based dose calculation itself from the small planned‐dose discrepancy already present between the LB plan dose and the LB measured dose. For HH‐iCBCT, the mean absolute difference between the calculated and measured dose across the 15 points was 2.61% (range: 0.8%–3.9%); for HH‐Acuros, 2.02% (range: 0.6%–3.3%); and for HH‐MAR, 1.69% (range: −0.1% to 4.3%, maximum at Lt_lung1). Notably, these calculated‐versus‐measured differences were similar to, or smaller than, the corresponding LB‐measured‐referenced differences reported above—particularly for HH‐MAR (1.69% vs. 2.90%)—indicating that CBCT‐based dose calculation accuracy itself is not the dominant source of the larger discrepancy previously observed for HH‐MAR relative to the LB measured reference.

**TABLE 3B:** TPS (Eclipse)‐calculated dose versus BeO OSLD‐measured dose at each of the 15 anatomical points, for each independently re‐optimized HH plan (unit: CGy).

	HH‐iCBCT			HH‐Acuros			HH‐MAR		
Point	Calc.	Meas.	Diff.(%)	Calc.	Meas.	Diff.(%)	Calc.	Meas.	Diff.(%)
**CTV1**	245.7	251.27 ± 1.82	2.3	243.3	249.47 ± 0.85	2.5	241.1	244.53 ± 2.29	1.4
**CTV2**	237.6	240.43 ± 0.71	1.2	239.6	244.80 ± 1.10	2.2	239.3	245.53 ± 0.85	2.6
**OAR1**	108.5	111.70 ± 0.72	3.2	109.5	113.10 ± 0.26	3.3	115.8	116.13 ± 0.21	0.3
**OAR2**	73.2	74.97 ± 0.42	2.4	73.2	74.37 ± 1.22	1.6	72.4	73.10 ± 0.46	1.0
**OAR3**	141.4	145.30 ± 0.72	2.8	138.5	139.37 ± 0.35	0.6	135.8	135.70 ± 0.70	−0.1
**OAR4**	73.3	75.03 ± 0.40	2.4	73.7	75.80 ± 1.14	2.8	71.7	72.43 ± 0.78	1.0
**OAR5**	46.2	47.13 ± 0.49	2.0	46.6	47.00 ± 0.17	0.9	49.0	49.40 ± 1.35	0.8
**OAR6**	40.9	42.33 ± 1.20	3.5	43.3	44.00 ± 0.75	1.6	41.3	41.30 ± 0.30	0.0
**OAR7**	42.9	44.57 ± 0.31	3.9	43.1	43.73 ± 0.35	1.5	45.3	46.63 ± 0.42	2.9
**Rt_lung1**	94.2	96.87 ± 0.57	2.8	95.3	98.23 ± 0.42	3.1	94.9	95.93 ± 0.51	1.1
**Rt_lung2**	97.5	100.03 ± 0.80	2.6	97.2	99.40 ± 0.62	2.3	97.7	100.97 ± 0.61	3.3
**Rt_lung3**	59.4	61.17 ± 0.83	3.0	62.7	63.77 ± 0.61	1.7	62.4	63.37 ± 0.70	1.5
**Lt_lung1**	78.6	81.47 ± 0.65	3.6	81.3	82.87 ± 1.63	1.9	78.6	82.00 ± 0.72	4.3
**Lt_lung2**	99.2	101.83 ± 0.65	2.7	99.1	102.33 ± 0.55	3.3	98.8	100.20 ± 0.10	1.4
**Lt_lung3**	80.7	81.33 ± 0.68	0.8	80.4	81.20 ± 0.87	1.0	75.9	78.73 ± 1.35	3.7

*Note*: Calc. = Eclipse‐calculated dose extracted from the saved dose distribution of each independently re‐optimized HH plan at the corresponding OSLD point location; Meas. = BeO OSLD‐measured dose (mean ± SD of three repetitions, reproduced from Table 3A); Diff.(%) = (Measured−Calculated) / Calculated × 100.

## 4 DISCUSSION

The present study extended prior work on HH CBCT by integrating TG‐233‐compliant task‐based image quality evaluation across all four insert materials, EPID‐based portal dosimetry, and multi‐point in vivo BeO OSLD dose verification in the RANDO phantom. Specifically, this study builds upon the work of Nelson et al.^20^ by incorporating direct multi‐point in vivo dose verification in an anthropomorphic phantom structure.

The statistically significant *d*′ values exceeding LB for HH‐iCBCT and HH‐Acuros indicate that iterative reconstruction—through integrated scatter correction and noise suppression—achieves sufficient signal‐to‐noise ratio (SNR) improvement to attain planning CT‐level clinical detectability. This is consistent with Lassot‐Buys et al.,^19^ who reported that iCBCT on Halcyon significantly improved *d*′ relative to FDK (referred to as FBP in that publication) and reduced noise magnitude by an average of 35% (head‐and‐neck), 19% (thorax), and 25% (pelvis). Notably, HH‐iCBCT maintained elevated *d*′ across all four insert materials, addressing a limitation of prior investigations that were restricted to single‐material analyses, and suggesting material‐independent robustness of this algorithm.^26^, ^27^

For HH‐MAR, the mean overall *d*′ (4.71 ± 0.08) was numerically the highest among HH algorithms and also showed a statistically significant improvement over the LB reference (*p* = 0.024), similar to HH‐iCBCT and HH‐Acuros.

In material‐specific analysis, HH‐MAR showed a notably elevated *d*′ for polyethylene (5.41, +24.6% vs. LB), but performed below the LB reference for acrylic (*d*′ = 4.31) and bone (*d*′ = 4.46), indicating material‐dependent variability. Furthermore, in vivo dose verification revealed that HH‐MAR yielded higher mean (2.90%) and maximum dose errors (−5.32% at Lt_lung2) than the other algorithms. Thus, although HH‐MAR achieved statistically significant detectability relative to LB, its comparatively larger in vivo dosimetric errors warrant caution, and additional validation—particularly incorporating an actual metal implant—is recommended before routine clinical deployment for its intended MAR indication. Lassot‐Buys et al.^19^ similarly cautioned that iterative reconstruction may complicate structure boundary delineation due to image smoothing; the MAR results here further highlight algorithm‐specific limitations.^28^ We emphasize that HH‐MAR was evaluated here under identical, metal‐free conditions as the other algorithms in order to characterize its baseline task‐based image‐quality and dose‐calculation behavior when applied outside its intended indication—a scenario of practical relevance because automated CBCT‐based ART workflows may apply MAR by default regardless of whether a metal implant is present. The present results should therefore be interpreted strictly as a metal‐free baseline characterization and not as a validation of HH‐MAR's intended artifact‐reduction performance; a dedicated evaluation incorporating an intentionally introduced metallic implant in the RANDO phantom, compared against the corresponding LB reference plan/image, is planned as a separate follow‐up study.

The finding that CBCT‐based dose calculation agreed with planning CT within 1% is consistent with Nelson et al.^20^ (≤ 1% in ion chamber measurements). Nelson et al.^20^ also reported that the HU‐to‐density calibration curve for HyperSight CBCT lies within 30 HU of the CT simulator for near‐water‐equivalent materials, while differences of up to 150 HU may occur for high‐density materials. To place the observed ΔHU values in dosimetric context, published sensitivity data for megavoltage photon‐beam dose calculation indicate that an approximately 8% error in bone relative electron density (RED) corresponds to roughly a 1% dose‐calculation error for typical treatment beams.^29^ The largest ΔHU observed among the HH algorithms (bone: Up to +69.2 HU for HH‐FDK, +47.5 to +62.8 HU for the other HH algorithms; polyethylene: +11.0 to +23.7 HU) corresponds to a relative HU deviation well below this ∼8% RED‐error threshold for typical bone‐equivalent CT numbers, implying an expected dose‐calculation impact of well under 1%, despite the use of a single uniformly applied calibration curve. In the present study, a single LB‐derived HU‐to‐ED calibration curve was intentionally applied to all CBCT algorithms to reflect realistic online ART workflows; however, this also represents a limitation in that algorithm‐specific calibrations were not employed. Duan et al.^12^ reported that HH‐iCBCT‐based dose calculation achieved a mean error within 1%–2% relative to the CT simulator in an online ART workflow. Furthermore, the multi‐institutional validation study by Lin et al.^13^ confirmed consistent HyperSight CBCT‐based dose calculation accuracy (gamma pass rate ≥ 95% at 1%/1 mm criterion) across diverse O‐ring linear accelerator environments. The portal dosimetry results in the present study (HH‐iCBCT: 95.0%; HH‐Acuros: 92.9%) align with these prior findings and provide complementary evidence confirming equivalent dose calculation reliability in a single‐institution thoracic VMAT setting.

In RANDO phantom BeO OSLD in vivo dose verification, mean dose errors for HH‐iCBCT (1.71%) and HH‐Acuros (2.09%) both satisfied the clinical quality assurance (QA) criterion of ± 5%. Given the OSLD measurement uncertainty of 1.6% (1σ) reported by Alvarez et al.,^22^ the observed 2%–3% dose differences likely reflect a combination of OSLD measurement uncertainty, algorithm‐specific CBCT HU differences, and air gaps between phantom slices. The BeO myOSLchip system used in this study (RadPro International GmbH / Freiberg Instruments GmbH) carries a manufacturer‐specified dose accuracy of ± 5%, consistent with the ± 5% clinical acceptance criterion adopted throughout this study. Beyond this manufacturer specification, an independent clinical validation of the same myOSLchip system, following the AAPM TG‐191 formalism (batch sensitivity correction, readout‐depletion correction, signal‐fading correction, and beam‐quality/angular correction), reported a combined dose‐measurement uncertainty of 3.3% (1.99 ± 0.06 Gy at a 2.0 Gy reference dose) under reference 6 MV conditions, with readout‐depletion of 1.6% per reading, signal‐fading of up to ∼1% for a typical batch readout duration, and negligible (< 3%) energy and angular dependence individually characterized.^30^ Taken together with the study‐specific Type A repeatability already observed across the three repeated OSLD measurements per point in the present work (SD < 1.5%), this combined ∼3.3% system‐level uncertainty is consistent with, and helps contextualize, the 2%–3% mean dose differences observed for HH‐iCBCT and HH‐Acuros. Indeed, Nelson et al.^20^ noted that the slice‐based design of the RANDO phantom can introduce air gaps that affect HU representation, potentially increasing discrepancies between imaging modalities. Mijnheer et al.^21^ reported that OSLDs maintain excellent reproducibility for cumulative doses up to 10 Gy, consistent with the repeated measurement results in this study (SD across 3 repetitions < 1.5%).

The elevated dose errors in the lung region, common across all algorithms, likely reflect the sensitivity of CBCT HU reconstruction to the low electron density and tissue heterogeneity of lung parenchyma. The maximum error for HH‐MAR (−5.32% at Lt_lung2) was greater than for HH‐iCBCT (−3.78%) and HH‐Acuros (−3.52%), highlighting the need for additional scrutiny of HH‐MAR dose accuracy in the lung. These findings are consistent with prior reports showing that dose calculation errors in low‐density heterogeneous regions such as the lung tend to exceed those in the CTV, owing to inherent limitations of CBCT HU reproducibility.^20^, ^26^

As Mijnheer et al.^21^ emphasized, in vivo dosimetry is essential for verifying three‐dimensional dose distributions that cannot be fully assessed by portal dosimetry alone, by directly confirming agreement between treatment planning system (TPS)‐calculated and delivered doses. In CBCT‐based online ART—where treatment decisions rely on same‐day imaging—an evaluation framework that integrates task‐based image quality metrics (d′, NPS, TTF) with multi‐point in vivo dose verification is particularly important for establishing the safety and reliability of clinical implementation.

The small sample size of *n* = 3 (df = 2) limits the statistical power of the one‐sample *t*‐test; Cohen's d, provided as a supplementary effect size measure in Table 1, should therefore be considered alongside p‐values. The large Cohen's d values for HH‐iCBCT (*d* = 4.67, *p* = 0.016) and HH‐Acuros (*d* = 9.50, *p* = 0.005) corroborate both the statistical significance and the practical clinical magnitude of these differences. HH‐MAR similarly showed a large Cohen's d (*d* = 3.63, *p* = 0.024), further corroborating the statistical significance of this improvement.^31^ Because the LB planning CT reference (*μ*_0_ = 4.42) was acquired/generated once and each of the six other reconstruction algorithms was compared against this single fixed reference using *n* = 3 technical replicates, each comparison in Table 1 is appropriately treated as a one‐sample *t*‐test against a fixed reference mean rather than a paired two‐sample test. As these six comparisons constitute a family of tests, a Holm‐Bonferroni step‐down correction^32^ (*α* = 0.05) was additionally applied to the reported *p*‐values: Ranked from smallest to largest (TB‐FDK and HH‐FDK, both *p* < 0.001; HH‐Acuros, *p* = 0.005; HH‐iCBCT, *p* = 0.016; HH‐MAR, *p* = 0.024; TB‐iCBCT, *p* = 0.035), the corresponding Holm‐adjusted significance thresholds are 0.0083, 0.0100, 0.0125, 0.0167, 0.0250, and 0.0500, respectively—all of which exceed the corresponding unadjusted *p*‐values, so that all six comparisons remain statistically significant after multiple‐comparison correction. We also wish to clarify explicitly, in response to the reviewer's underlying concern, that these *n* = 3 acquisitions are technical replicates of the same static phantom under fixed setup conditions rather than independent clinical samples (e.g., different patients, fractions, or anatomical presentations). Accordingly, the statistical significance reported here demonstrates a reproducible measurement difference under fixed technical conditions and should not be interpreted as evidence of population‐level (inter‐patient) generalizability, which this phantom‐based design cannot, by itself, establish; this distinction is now also noted explicitly in the Limitations paragraph below.

This study has several limitations. First, a single LB‐derived HU‐to‐ED calibration curve was applied to all CBCT algorithms, which may not fully account for algorithm‐specific HU differences; future studies should employ individually optimized HU‐to‐density calibration curves for each algorithm, as recommended by Nelson et al.^20^ Second, the *n* = 3 acquisitions per algorithm are technical replicates obtained from a single static phantom setup rather than independent clinical samples (e.g., different patients or treatment fractions); the statistically significant differences reported therefore reflect reproducible measurement differences under fixed technical conditions and should not be interpreted as directly generalizable to inter‐patient variability, which a phantom study cannot, by itself, establish. Third, as a phantom‐based study, physiological variables such as respiratory motion and organ displacement were not simulated, and inter‐slice air gaps in the RANDO phantom may partly increase measurement uncertainty. Fourth, BeO OSLD measurements at 15 discrete points cannot fully characterize the three‐dimensional dose distribution. Patient‐based prospective studies across diverse anatomical sites and algorithm‐specific calibration investigations are needed.^33^ Fifth, findings from a single‐institution, single thoracic VMAT plan may not generalize broadly; additional verification across other anatomical sites (e.g., head‐and‐neck, pelvis) and treatment modalities is warranted.

## 5 CONCLUSIONS

This study evaluated the Halcyon HyperSight imaging platform and its FDK, iCBCT, Acuros, and MAR reconstruction algorithms using an integrated approach that encompassed ImQuest software‐based task‐based image quality assessment compliant with TG‐233 across all four insert materials, Eclipse‐based dose calculation, and in vivo BeO OSLD dose verification in the RANDO anthropomorphic phantom. Our results indicated that HH‐iCBCT (*d*′ = 4.70, *p* = 0.016) and HH‐Acuros (*d*′ = 4.61, *p* = 0.005) demonstrated statistically significant improvements in detectability relative to LB planning CT, while HH‐iCBCT showed the most consistent image quality performance across all four insert materials. CBCT‐based dose calculations yielded errors within 1% of LB planning CT across all algorithms. In RANDO phantom BeO OSLD verification, HH‐iCBCT (mean error 1.71%) and HH‐Acuros (mean error 2.09%) met the clinical acceptance criterion of ± 5% at all 15 measurement points, whereas HH‐MAR (mean error 2.90%) exceeded the tolerance limit at the Lt_lung2 point (−5.32%). Overall, these findings support the use of HH‐iCBCT as a clinically practical and well‐balanced reconstruction algorithm for both image guidance and online dose calculation in CBCT‐based adaptive radiotherapy (ART) workflows.

## AUTHOR CONTRIBUTIONS

Byungmoon Park and Kwan‐Woo Lee conceived and designed the study and wrote the manuscript. Byungmoon Park and Taeyoon Kim performed the phantom measurements, image quality analysis, and dosimetric verification. Byungmoon Park performed the statistical analysis. All authors analyzed and discussed the results, reviewed the manuscript, and approved the final version.

## CONFLICT OF INTEREST STATEMENT

The authors declare that they have no known competing financial interests, personal relationships, or affiliations with commercial entities (including Varian Medical Systems or Duke University) that could have appeared to influence the design, conduct, analysis, or interpretation of the work reported in this paper.

## ETHICAL APPROVAL

This study was conducted entirely using anthropomorphic and physical phantoms (Gammex 464 ACR phantom and Alderson RANDO phantom); no human participants or animal subjects were involved at any stage of data acquisition or analysis. Accordingly, institutional review board (IRB) approval and informed consent were not required for this work, in accordance with institutional policy.

## USE OF ARTIFICIAL INTELLIGENCE TOOLS

An AI‐based language model (Claude, Anthropic) was used to assist with language editing of the manuscript and to generate figures based on the measured data. All scientific content, data analysis, and interpretations were performed and verified by the authors.

## Data Availability

The raw and processed data supporting the findings of this study, including ImQuest‐derived image quality metrics, Eclipse dose calculation outputs, and BeO OSLD measurement records, are available from the corresponding author upon reasonable request. The data are not publicly deposited due to institutional data‐sharing restrictions.
